# Case Report: A steroid-sparing mechanical management of IgG4-related sclerosing cholangitis mimicking cholangiocarcinoma

**DOI:** 10.3389/fimmu.2026.1803019

**Published:** 2026-04-23

**Authors:** Zhenchao Liu, Aihua Wang, Yu Cao, Xinbo Zhao

**Affiliations:** 1 School of Pharmacy, Qingdao University, Qingdao, China; 2 Department of Gastroenterology, Linyi People’s Hospital, Linyi, China; 3 Clinical Trials Center, The Affiliated Hospital of Qingdao University, Qingdao, China; 4 Department of Endocrinology, Linyi People’s Hospital, Linyi, China

**Keywords:** CA19-9, case report, cholangiocarcinoma, IgG4, IgG4-SC

## Abstract

**Background:**

IgG4-SC typically requires long-term corticosteroid therapy. We report a distinctive case of a 65-year-old woman with biopsy-supported IgG4-SC that closely mimicked malignancy.

**Case summary:**

The patient presented with 10 kg weight loss and CA19–9 of 315 U/mL. She achieved sustained clinical and biochemical remission after repeated endoscopic mechanical decompression alone, with no recurrent obstructive cholestasis during long-term clinical and biochemical follow-up. However, serum IgG4 remained elevated at long-term reassessment, and complete immunologic remission could not be assumed.

**Conclusion:**

This case raises the possibility of an “obstruction-dominant” presentation of IgG4-SC and suggests that intensive endoscopic biliary decompression may serve as a temporary or, in selected cases, sustained steroid-sparing strategy in carefully selected high-risk patients, without challenging the current role of corticosteroids as standard first-line therapy.

## 1 Introduction

IgG4-related sclerosing cholangitis (IgG4-SC) is the biliary manifestation of IgG4-related disease (IgG4-RD), a systemic, immune-mediated fibroinflammatory disorder characterized by tumefactive lesions, a dense lymphoplasmacytic infiltrate rich in IgG4-positive plasma cells, and characteristic “storiform” fibrosis (1, 2). Epidemiologically, Japanese nationwide surveys indicate that IgG4-SC exhibits its highest incidence in individuals aged 60–80 years (3), a demographic often burdened with metabolic comorbidities. Since its initial recognition, IgG4-SC has emerged as an important diagnostic challenge in hepatobiliary medicine due to its remarkable ability to mimic malignant biliary strictures, such as perihilar or distal cholangiocarcinoma (CCA) and pancreatic head carcinoma (4, 5).

This is underscored by the fact that approximately 15–20% of patients undergoing radical surgery for presumed biliary malignancy are postoperatively diagnosed with benign inflammatory conditions, with IgG4-SC representing an important benign cause (2, 4). This mimicry is not limited to imaging: elevated carbohydrate antigen 19-9 (CA19-9), profound weight loss, and progressive jaundice-traditionally considered “red flags” for malignancy-are frequently observed (6). The immunopathogenesis is thought to involve a Th2-dominant immune response in which cytokines contribute to both IgG4 subclass switching and the fibrotic response (2). Recent insights (2, 4) suggest that chronic biliary irritation may act as a potential “antigenic trigger” that remains under-explored (7).

Current international guidelines advocate for systemic glucocorticoids as the first-line treatment (8). However, long-term steroid use carries significant morbidity, especially in elderly populations with diabetes. To our knowledge, only a limited number of English-language reports have described biopsy-supported IgG4-SC achieving sustained clinical and biochemical remission with serial endoscopic mechanical management alone, without corticosteroid therapy. We present a unique case of biopsy-proven IgG4-SC in which sustained clinical and biochemical remission was achieved through serial mechanical endoscopic decompression alone, without corticosteroid or immuno-suppressive therapy. By detailing this clinical course, we raise the possibility of an “obstruction-dominant” presentation, in which relief of biliary stasis may have contributed to attenuation of the inflammatory process.

## 2 Case description

### 2.1 Patient information

A 65-year-old Han Chinese woman presented to our center in June 2020 with a 4-day history of progressive obstructive jaundice, characterized by scleral icterus, dark urine, acholic stools, and debilitating pruritus. Notably, the patient reported a significant unintended weight loss of 10 kg over the preceding six months. Her medical history included insulin-dependent type 2 diabetes (HbA1c 8.2%), hypertension (well-controlled on amlodipine), and depression (3-year history). She was a lifelong non-smoker and abstained from alcohol, with no family history of hepatobiliary malignancy or autoimmune diseases.

### 2.2 Physical examination and clinical findings

On physical examination, the patient was afebrile but markedly icteric. Abdominal examination was unremarkable, with no palpable masses, organomegaly, or tenderness. Initial laboratory evaluation confirmed a severe cholestatic pattern: total bilirubin (TBIL) was 100 μmol/L (direct bilirubin 88.5 μmol/L), ALT 96 U/L (reference range, 9–50 U/L), AST 79 U/L (reference range, 15–40 U/L), ALP 287 U/L (reference range, 40–130 U/L), and GGT 1176 U/L (reference range, 10–60 U/L). Tumor markers revealed a significantly elevated CA19–9 level of 315 U/mL (normal <37 U/mL) and a slightly elevated CEA of 5.4 μg/L.

### 2.3 Ethics and consent

This study was approved by the Science and Technology Ethics Committee of Linyi People’s Hospital (NO.202504-H-011). Written informed consent was obtained from the patient for the publication of this case report and accompanying images.

## 3 Timeline

The patient’s clinical course is summarized in **Table 1**, which outline the dates of each ERCP, bilirubin changes, serum IgG4 and CA19–9 trends, stent management, and follow-up assessments.

**Table 1:** Timeline of clinical events, diagnostic findings, and therapeutic interventions.

Date	Clinical milestone/indication	Key indicators & imaging results	Therapeutic intervention (procedural details)	Pathology/cytology	Immediate outcome/safety
6-Jun-2020	Initial presentation with fatigue and jaundice	TBIL 100 μmol/L; DBIL 88.5 μmol/L; ALT 96.2 U/L (reference 9–50); AST 79.7 U/L (reference 15–40); ALP 287 U/L (reference 40–130); GGT 1176 U/L (reference 10–60); CA19-9 315.1 U/mL	Admitted for obstructive jaundice of unknown origin; supportive treatment initiated (glutathione, compound glycyrrhizin, nutritional/supportive care)	—	Jaundice persisted; no procedure-related adverse event
8-10 Jun-2020	Diagnostic reassessment for suspected malignant obstruction	CT/MRI/MRCP: distal common bile duct (CBD) stricture, biliary dilatation, pancreatic head/uncinate fullness suspicious for pancreatic malignancy	Surgical evaluation performed; operative treatment discussed but declined by family	—	Malignancy strongly suspected clinically
15-Jun-2020	1st ERCP for biliary decompression of distal CBD obstruction	Cholangiography: proximal CBD dilated to ~1.8 cm; distal CBD stenosis	ERCP + EST + brush cytology + forceps biopsy + placement of uncovered SEMS (6 cm × 1 cm) + ENBD for drainage	Bile duct brush cytology: reactive biliary epithelial cells; no definite malignancy	Bile drained well with immediate decompression; no post-ERCP pancreatitis, bleeding, perforation, or cholangitis
19-Jun-2020	Clinical worsening after initial drainage; reassessment of stent patency/persistent obstruction	TBIL increased to 145.2 μmol/L; AST 413.3 U/L; ALT 239.5 U/L; ALP 455 U/L; GGT 914 U/L	2nd ERCP: repeat cholangiography confirmed persistent distal CBD narrowing; repeat brushing/biopsy and biliary drainage optimization through previously placed metallic stent with nasobiliary drainage	Repeat sampling nondiagnostic for malignancy	Biliary outflow re-established; no immediate procedural complication
25-Jun-2020	Early post-drainage reassessment	TBIL decreased to 44.2 μmol/L; DBIL 37.5 μmol/L; GGT 247 U/L	Discharged with biliary stent in place; referred externally because pancreatic malignancy was still suspected	—	Clinical improvement of jaundice
Jul-2020	Diagnostic shift begins	Serum IgG4 14.70 g/L (reference <1.35 g/L), markedly elevated	Re-evaluation of presumed malignancy; steroid therapy intentionally deferred because cancer had not yet been confidently excluded	—	IgG4-RD/IgG4-SC entered differential diagnosis
6-Aug-2020	Follow-up after decompression and external reassessment	CA19-9 normalized to 23.9 U/mL; PET/CT showed no definite hypermetabolic malignancy	Continued surveillance; decision made to reassess biliary tract endoscopically	—	Suspicion for cancer decreased substantially
10-Aug-2020	3rd ERCP for stent management after diagnostic reconsideration	Cholangiography: CBD only mildly dilated (~1.2 cm); previously placed uncovered SEMS visible and difficult to remove	ERCP + biopsy; attempted removal of uncovered SEMS failed with snare/forceps; a covered SEMS (8 cm × 1 cm) was placed coaxially to maintain drainage and facilitate later extraction	Bile duct biopsy obtained	Bile drainage remained good; no immediate complications
9-13 Sep-2020	4th-5th ERCPs for removal of retained uncovered SEMS	Persistent exposed metallic stent; biliary drainage maintained	4th ERCP: covered SEMS removed successfully, but original uncovered SEMS could not be extracted. 5th ERCP: APC used to trim/cut embedded uncovered SEMS, which was then removed endoscopically; a 7F × 5 cm double-pigtail plastic stent was inserted for short-term drainage	Papillary biopsy: tubular adenoma with low-grade intraepithelial neoplasia, focal area suspicious for high-grade change	Successful extraction of embedded metallic stent; no perforation, major bleeding, pancreatitis, or cholangitis reported
30-Sep-2020	6th ERCP for persistent distal CBD narrowing and duct clearance	Cholangiography: upper CBD ~1.2 cm, distal segment thin/narrow; no mass proven	ERCP + balloon sweep/clearance + biopsy + placement of covered SEMS (10 mm × 6 cm); debris/sludge and fragmented microlithiasis-like material were removed from the bile duct	Papillary biopsy: chronic inflammation with infection and fibrous tissue hyperplasia; no malignancy	Duct clearance achieved and biliary drainage improved; no immediate complications
30-Nov-2020	Intermediate follow-up	IgG4 3.35 g/L; CT: no pancreatic mass, pancreatic atrophy only; no recurrent jaundice	Continued observation without steroids or immunosuppressants	—	Sustained biochemical and radiologic improvement
5-Mar-2021	7th ERCP for planned stent removal and final reassessment	TBIL 5.3 μmol/L; DBIL 2.5 μmol/L; liver enzymes normalized; IgG4 2.80 g/L	ERCP + removal of residual stent + balloon extraction of small CBD stones + repeat biopsy + ENBD; cholangiography showed only mild CBD dilatation (~1.2 cm) with small filling defect, cleared by balloon extraction	Papillary biopsy: chronic active mucosal inflammation with abundant plasma-cell infiltration; immunostaining showed IgG-positive plasma cells and scattered IgG4-positive plasma cells, supporting IgG4-related disease in the overall clinicopathologic context	All stents removed; complete biliary drainage restored; no recurrence of jaundice and no ERCP-related major adverse events during follow-up
Dec-2021 (approximately 9 months after final ERCP)	Short-to-intermediate post-procedural follow-up	Patient asymptomatic; liver biochemistry stable; follow-up imaging reportedly showed no recurrent biliary stricture or mass lesion	Observation only	—	No clinical relapse documented
14-Mar-2026	Extended outpatient follow-up	TBIL 9.2 μmol/L (reference 2–24); DBIL 2.0 μmol/L (reference 0–3.4); ALT 13.9 U/L (reference 7–40); AST 19.6 U/L (reference 13–35); ALP 144.1 U/L (reference 40–150); GGT 16.7 U/L (reference 7–45); CRP <3.1 mg/L (reference 0–8); IgG4 6.67 g/L (reference 0.03–2.1). Additional metabolic/renal parameters: glucose 9.39 mmol/L, creatinine 112.0 μmol/L, UACR 94.9 mg/g	Outpatient clinical and laboratory reassessment only; no corticosteroids or immunosuppressive therapy given	—	No recurrent jaundice, pruritus, abdominal pain, or other symptoms of biliary obstruction; long-term clinical and biochemical remission maintained, although serum IgG4 remained elevated

## 4 Diagnostic assessment, therapeutic intervention, follow-up, and outcomes

### 4.1 Diagnostic assessment

**Figure 1:**
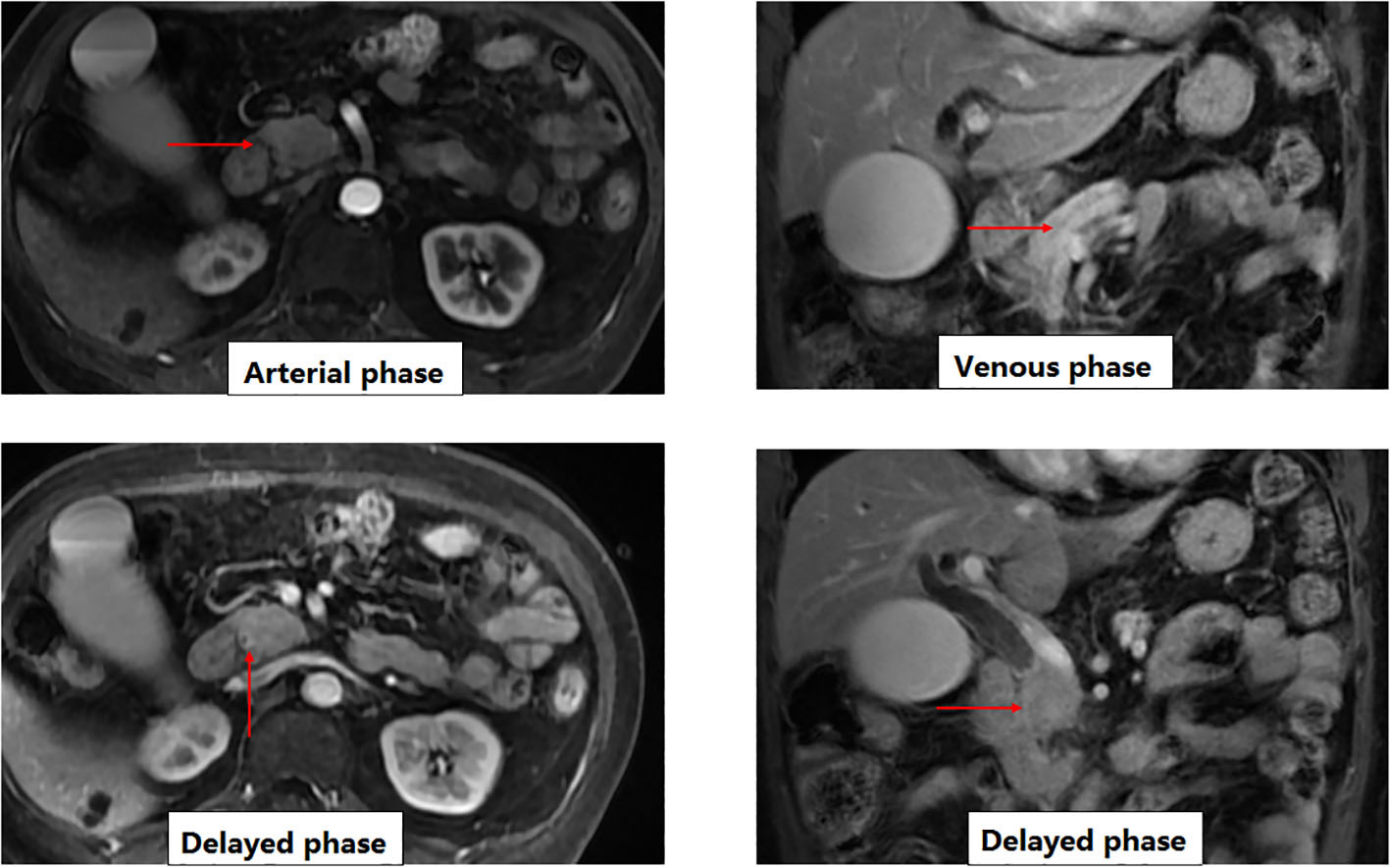
Dynamic contrast-enhanced MRI findings of the distal common bile duct and pancreatic head show a soft tissue mass at the distal common bile duct (red arrow), exhibiting relatively low enhancement in the arterial phase. In the venous phase, the lesion demonstrates moderate, gradual enhancement (red arrow). During the delayed phase, the lesion shows persistent and progressive enhancement (red arrows), a radiologic pattern that may be seen in fibroinflammatory lesions such as IgG4-related sclerosing cholangitis and that, in this case, supported further tissue-based evaluation. Although these findings did not exclude malignancy, the delayed progressive enhancement pattern raised the possibility of a fibroinflammatory process and supported further tissue-based evaluation.

Initial diagnostic evaluation focused on contrast-enhanced MRI to characterize the biliary obstruction. Multi-phase imaging revealed an approximately 2.5 cm smooth-walled stricture in the distal common bile duct (CBD), characterized by significant concentric mural thickening (See **Figure 1**). A key radiologic feature was the dynamic enhancement pattern: the thickened CBD wall and the mildly lobulated pancreatic head showed relatively low enhancement during the arterial phase, followed by progressive, persistent enhancement in the venous and delayed phases. This delayed progressive enhancement pattern raised the possibility of a fibroinflammatory process such as IgG4-related disease, although it was not by itself sufficient to exclude malignancy.

**Figure 2:**
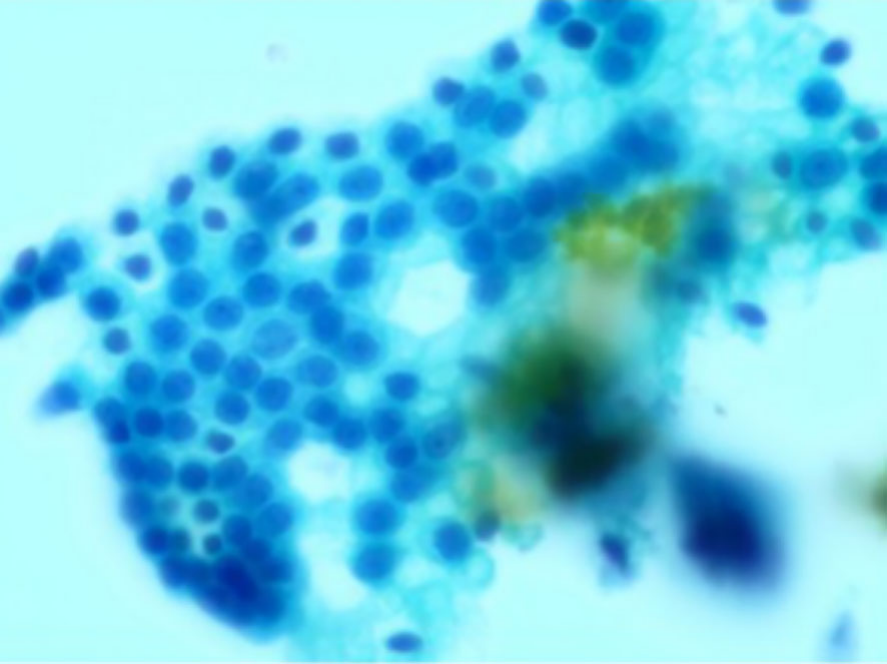
Initial biliary brush cytology: Cytology from the first ERCP procedure (Papanicolaou stain, 400x magnification) showing clusters of biliary epithelial cells with slight architectural irregularity, enlarged nuclei, and prominent nucleoli. These features were interpreted as reactive epithelial changes secondary to inflammation/obstruction rather than evidence of malignancy, providing an early but inconclusive clue toward a benign process.

To further differentiate the lesion, initial biliary brush cytology was performed during the first ERCP. The cytological analysis suggested reactive epithelial atypia in the setting of obstruction/inflammation, but it was not sufficient by itself to exclude cholangiocarcinoma. (See **Figure 2**).

The principal differential diagnosis was distal cholangiocarcinoma, given the patient’s weight loss, obstructive jaundice, distal CBD stricture, and elevated CA19-9. However, the absence of a definite mass with malignant imaging characteristics, only mild FDG uptake on PET/CT, nondiagnostic brush cytology, marked serum IgG4 elevation, and subsequent histology from ERCP-guided biopsy favored IgG4-SC over malignancy. Autoimmune pancreatitis with secondary biliary involvement was also considered because of the mildly lobulated pancreatic head on MRI; however, the available pancreatic imaging and clinical findings were insufficient to support a definite diagnosis of type 1 autoimmune pancreatitis. Primary sclerosing cholangitis was considered less likely because the lesion was focal and distal rather than multifocal, the patient lacked a history of inflammatory bowel disease, and the overall clinical, serologic, and histologic profile was more consistent with IgG4-SC. Thus, the final diagnosis was based on the cumulative clinicopathologic pattern rather than on any single test in isolation.

Subsequent PET/CT scan in August 2020 further supported this, showing only mild inflammatory uptake at the distal CBD (SUVmax 3.2) without evidence of distant metastasis. Importantly, serum IgG4 was not obtained before the initial ERCP. It was measured during the subsequent diagnostic work-up after initial ERCP-based decompression and was markedly elevated at 14.7 g/L (normal <1.35 g/L). All diagnostic tissue samples in this case were obtained from the distal common bile duct stricture during ERCP using deep intraductal “bite-on-bite” forceps biopsy; no pancreatic biopsy or surgical specimen was obtained. All pathology described in this report was derived from intraductal bile duct biopsies obtained during ERCP; no pancreatic biopsy or surgical specimen was available. Multiple samples were collected across separate procedures because the initial brush cytology was nondiagnostic and malignancy remained a major concern. Histopathologic review of the ERCP-guided bile duct biopsy specimens at an external referral center showed a dense lymphoplasmacytic infiltrate with focal storiform fibrosis. Immunohistochemistry demonstrated >10 IgG4-positive plasma cells per high-power field (exact peak count was not available in the referral pathology report) and an IgG4/IgG ratio >40%, findings consistent with IgG4-related sclerosing cholangitis in the appropriate clinicopathologic context. The original histology image files from the external institution were not retrievable for publication, which is a limitation of this report. Distal cholangiocarcinoma remained an important consideration throughout the diagnostic work-up. However, repeated ERCP-based cytology and intraductal biopsies did not demonstrate malignancy, serum IgG4 was markedly elevated, histology was consistent with IgG4-RD, cholestatic biochemistry improved after drainage, and the patient experienced a prolonged recurrence-free course without oncologic treatment; taken together, these findings made an occult aggressive malignancy increasingly unlikely.

### 4.2 Therapeutic intervention

In brief, the patient underwent seven ERCP procedures between June 2020 and March 2021, with initial decompression, subsequent management of debris-related stent occlusion, serial stent exchanges/clearance, and eventual complete stricture resolution permitting final stent removal.

A diagnostic steroid trial was not pursued because malignancy had not yet been confidently excluded at presentation, and the patient also had important relative contraindications to systemic corticosteroids, including insulin-dependent diabetes with suboptimal glycemic control and a history of depression.

Mechanical-first endoscopic approach. Between June 2020 and March 2021, the patient underwent a total of seven ERCP procedures, reflecting a purely mechanical treatment strategy.

1. Primary Decompression: Biliary sphincterotomy, balloon dilation of the distal CBD stricture, and placement of an uncovered self-expanding metal stent (SEMS). An uncovered SEMS was initially selected because malignant distal biliary obstruction had not yet been confidently excluded at the time of first intervention.
2. Management of Recurrent Obstruction: Following a post-stenting bilirubin flare (TBIL 145 μmol/L), subsequent ERCPs demonstrated stent occlusion by thick proteinaceous biliary debris and microlithiasis, requiring repeat endoscopic intervention. We intentionally avoid the nonstandard term “IgG4-sludge” and instead describe the endoscopic finding by its observed components.
3. Serial Clearance and Stent Exchange: Over the following nine months, five additional ERCPs were performed for repeat cholangiography, balloon clearance of proteinaceous debris/microlithiasis, and stepwise stent exchange or removal according to recurrent cholestasis, stent patency, and cholangiographic improvement of the distal CBD stricture. These repeated interventions were undertaken to maintain biliary drainage and manage debris-related stent occlusion. By the seventh ERCP (March 2021), the stricture had resolved both visually and fluoroscopically, allowing permanent stent removal.

No corticosteroids or immunosuppressive agents were administered throughout the course. No procedure-related major adverse events, including post-ERCP pancreatitis, clinically significant bleeding, perforation, or procedure-related cholangitis, were documented during the treatment course. Other than transient post-procedural bilirubin fluctuation related to stent occlusion, no major procedure-related adverse events were documented.

### 4.3 Follow-up and outcomes

**Figure 3:**
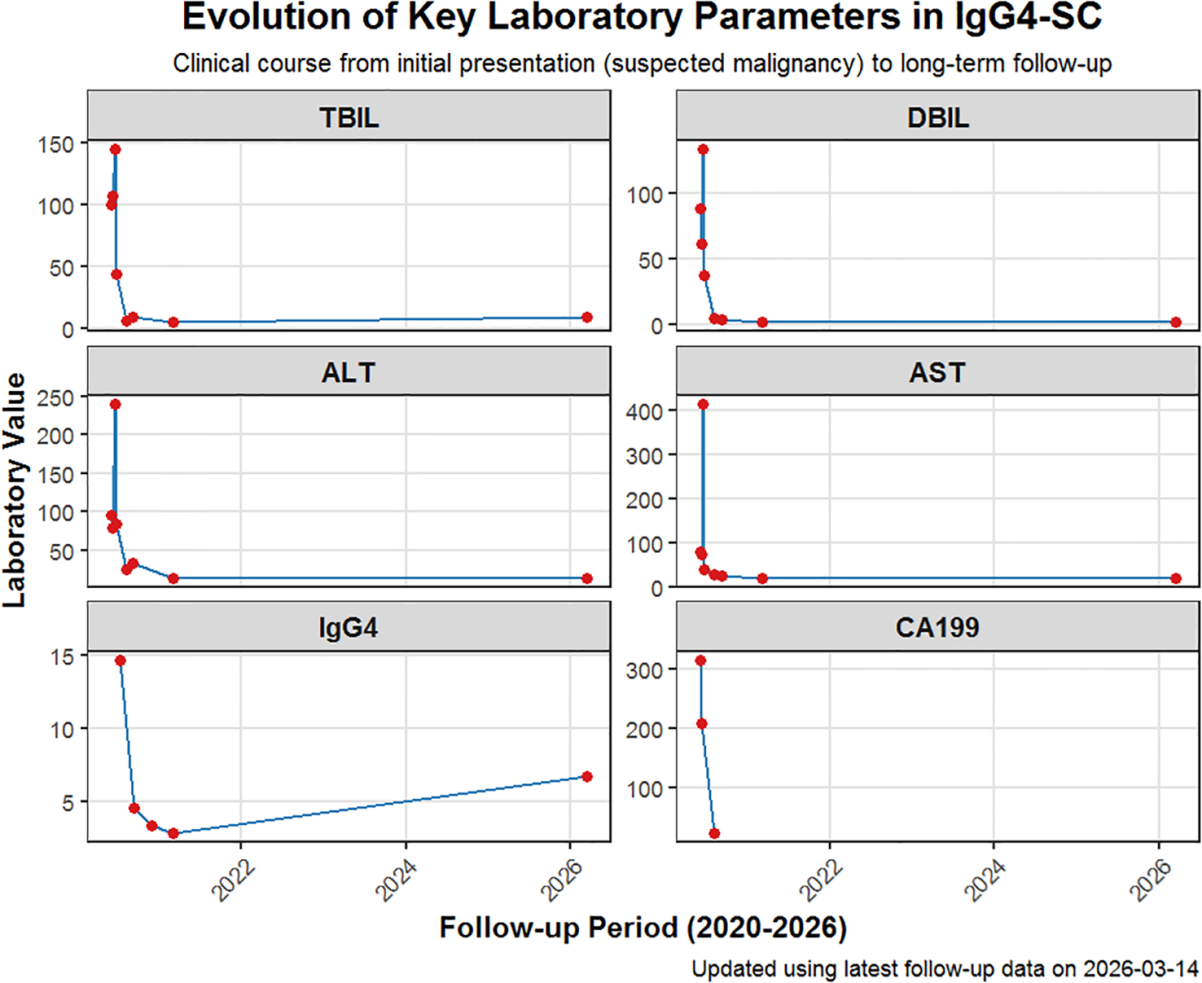
Serial changes in bilirubin, liver enzymes, CA19-9, and serum IgG4 during repeated endoscopic management.

Following restoration of ductal patency and removal of the final stent, the patient’s cholestatic biochemistry normalized without corticosteroid therapy. Serum IgG4 levels declined from 14.7 g/L to 3.35 g/L and then to 2.80 g/L by the final procedural follow-up in March 2021. CA19–9 and liver enzymes also returned to normal ranges. At 9 months after the final intervention, the patient remained asymptomatic, liver biochemistry was stable, and follow-up cross-sectional imaging showed no recurrent biliary stricture or mass lesion. Additional long-term outpatient clinical follow-up was available through March 14, 2026 (approximately 5 years and 9 months after presentation and nearly 5 years after final stent removal). At that reassessment, the patient remained free of recurrent jaundice, pruritus, abdominal pain, or other symptoms of biliary obstruction. Liver biochemistry was largely normal, with normal bilirubin, transaminases, and GGT; ALP was only minimally above the local reference range (144.1 U/L; reference range 40–130 U/L), and CRP was normal (<3.1 mg/L). Serum IgG4, however, remained elevated at 6.67 g/L (reference range 0.03–2.1 g/L). No repeat CT or MRCP was obtained at that visit. The differing upper reference limits for serum IgG4 reported in this case reflect testing performed at different laboratories over time (<1.35 g/L initially vs 0.03–2.1 g/L at long-term reassessment); values are reported as originally provided by the respective laboratories. Overall, these findings support sustained long-term clinical and biochemical remission without recurrent obstructive cholestasis; however, complete immunologic or radiologic remission cannot be confirmed, particularly because serum IgG4 remained elevated and no repeat CT/MRCP was obtained at the latest follow-up visit. (See **Figure 3**).

## 5 Discussion

### 5.1 The “great mimicker” and the CA19–9 diagnostic pitfall

IgG4-SC remains among the most diagnostically challenging conditions in hepatobiliary medicine due to its pronounced tendency to mimic malignant biliary diseases (8, 9). Our patient presented a classic “clinical trap”: a 2.5 cm distal CBD stricture, profound weight loss (10 kg), and a ten-fold elevation of CA19-9 (315 U/mL). Traditionally, CA19–9 levels exceeding 100 U/mL in the presence of a biliary stricture carry a high positive predictive value for malignancy (6, 10). However, this case reinforces that CA19–9 is often a marker of biliary epithelial stress rather than a tumor-specific antigen (5, 6).

As noted by Ventrucci et al. (5), CA19–9 elevation in benign IgG4-related biliary disease can reach levels traditionally diagnostic of terminal cholangiocarcinoma. The massive lymphoplasmacytic infiltration and subsequent storiform fibrosis exert significant extrinsic and intrinsic pressure on the biliary epithelium, triggering the over-expression and shedding of CA19–9 into the circulation via increased epithelial permeability (11, 12). The rapid, steroid-free normalization of CA19–9 in our patient following mechanical decompression underscores the importance of interpreting tumor markers in the context of ductal patency.

### 5.2 Considering an “obstruction-dominant” presentation

A major conceptual implication of this report is the possibility that some patients with IgG4-SC may present with a relatively obstruction-dominant clinical pattern. While the current HISORt and Japanese Biliary Association (JBA) criteria provide a robust framework for identification (8), they do not fully account for phenotypic heterogeneity in treatment response. As a hypothesis-generating conceptual framework based on this case and the recognized clinical heterogeneity of IgG4-SC, some presentations may perhaps be viewed heuristically along a spectrum:

1. Systemic/Inflammation-Dominant Phenotype: Characterized by multi-organ involvement (e.g., autoimmune pancreatitis) and rapid response to systemic corticosteroids.
2. Obstruction-Dominant Phenotype (The present case): Characterized by localized biliary involvement where mechanical factors-stasis, proteinaceous sludge, and microlithiasis-become the primary drivers of localized and systemic inflammation.

In our patient, the absence of overt systemic involvement and the favorable response to serial mechanical intervention raise the possibility that biliary obstruction itself may have played an important role in sustaining the local inflammatory process.

### 5.3 A hypothesis of mechanical-immune cross-talk

The reason serum IgG4 declined substantially after mechanical decompression alone remains uncertain. We propose a hypothesis of “mechanical-immune cross-talk.” Biliary stasis leads to the accumulation of Pathogen-Associated Molecular Patterns (PAMPs) and Damage-Associated Molecular Patterns (DAMPs) from stressed cholangiocytes (13). These molecules act as ligands for Toll-like Receptors (TLRs) on resident macrophages and dendritic cells. Chronic TLR activation may sustain the Th2 and Treg cell-mediated immune response, driving IL-10 and TGF-β production, which are essential for IgG4 subclass switching and fibrogenesis (2, 13–16).

Relief of obstruction through repeated ERCPs may have reduced the burden of secondary inflammatory or antigenic stimuli within the biliary tree, but this remains inferential and cannot be established from a single observational case. By clearing proteinaceous biliary debris and microlithiasis, repeated ERCP might have contributed to disrupting a putative cycle of stasis, epithelial injury, and ongoing immune activation. One speculative possibility is that relief of biliary obstruction reduced cholangiocyte injury and secondarily altered local immune signaling. Because biliary epithelial cells can function as non-professional antigen-presenting cells, decompression might theoretically modulate pathways such as HLA-DR expression and downstream immune activation (13, 17, 18). However, these possibilities remain hypothesis-generating and cannot be inferred as causal from a single case.

### 5.4 Clinical implications: a steroid-sparing paradigm

Systemic corticosteroids are the cornerstone of IgG4-SC management, yet they pose significant risks for elderly patients with comorbidities (8, 19). Our patient, with insulin-dependent diabetes and depression, was a high-risk candidate for steroid therapy. Glucocorticoids can cause severe glycemic dysregulation and exacerbate psychiatric conditions (8). Our experience suggests a possible management approach for selected high-risk patients in whom corticosteroids are undesirable or need to be deferred. By prioritizing ductal patency and systematic clearance of the biliary tree, sustained clinical and biochemical remission was achieved in this patient without pharmacologic immunosuppression. However, this observation should be interpreted cautiously and not as evidence that mechanical therapy can routinely replace corticosteroids. This case suggests that, in carefully selected high-risk patients, biliary drainage and repeated endoscopic clearance may play a more substantial therapeutic role than is usually emphasized, although current guidelines continue to support corticosteroids as standard first-line therapy (8).

### 5.5 Comparative analysis and clinical contextualization

As reported by Zhang et al. (4), IgG4-SC remains a primary cause of “unnecessary” major surgeries. Many patients with high-grade strictures undergo radical pancreaticoduodenectomy (the Whipple procedure) because their clinical profiles are indistinguishable from malignancy (4). Our case directly addresses this clinical trap; despite an aggressive presentation, we utilized the diagnostic window provided by repeated ERCP to avoid irreversible surgery.

Furthermore, unlike the patients in the study by Ventrucci et al. (5) who often required steroids to achieve biochemical normalization, our patient’s markers normalized in direct lockstep with mechanical resolution. This case is notable for three reasons: (1) the prolonged steroid-free follow-up, now extending to long-term outpatient reassessment; (2) the marked decline in an initially very high serum IgG4 level after serial mechanical intervention; and (3) the close temporal association between repeated biliary clearance and subsequent biochemical improvement. Nevertheless, the persistent elevation of serum IgG4 on long-term follow-up suggests that the observed remission is best interpreted as clinical and biochemical rather than complete immunologic remission.

### 5.6 Limitations and future directions

This report has several important limitations. First, it describes a single case and therefore cannot establish causality, generalizability, or a practice-changing treatment strategy. Second, although longer-term clinical and biochemical follow-up through March 2026 is reassuring, relapse remains possible, as is well recognized in IgG4-RD. Third, occult malignancy can never be excluded with absolute certainty. Distal cholangiocarcinoma was considered carefully throughout the work-up, but the combination of nondiagnostic malignant cytology/biopsy across repeated ERCP-based sampling, markedly elevated serum IgG4, histology supportive of IgG4-RD, biochemical improvement after drainage, and the prolonged recurrence-free clinical course without oncologic treatment made an occult aggressive malignancy progressively less likely. Finally, the latest long-term reassessment did not include repeat CT or MRCP, which limits the strength of radiologic confirmation of persistent remission.

Limitations of this observation include its single-case nature and the presence of gallbladder calculi. The latter serves as a potential confounder, as intermittent stone-related obstruction or chronic biliary irritation may have influenced the local immune profile and contributed to both local inflammation and debris formation. However, this report demonstrates that static imaging and single time-point serology are insufficient. Practical steps for clinicians include obtaining adequate deep-tissue biopsy to support clinicopathologic diagnosis within established diagnostic frameworks such as HISORt, and scheduling biomarker reassessment after obstruction relief (20). Future research should focus on establishing phenotype-based algorithms to distinguish obstruction-dominant from immune-predominant IgG4-SC, potentially sparing a subset of patients from the side effects of long-term immunosuppression.

Accordingly, the proposed “obstruction-dominant” presentation and the mechanical-immune hypothesis should be considered hypothesis-generating and require validation in larger cohorts.

## 6 Conclusion

This case suggests that, in highly selected patients with IgG4-SC, sustained clinical and biochemical remission may be observed after intensive mechanical biliary decompression without immediate corticosteroid therapy. Such an approach should be viewed only as an individualized, hypothesis-generating steroid-sparing consideration in patients with substantial comorbidity or relative contraindications to steroids. The proposed “obstruction-dominant” presentation should be regarded as a hypothesis-generating clinical construct rather than a validated disease subtype, and it should not be interpreted as challenging current guideline-supported first-line corticosteroid therapy.

## 7 Patient perspective

The patient expressed significant relief upon achieving remission without the need for long-term steroid therapy, which she had initially feared due to her history of insulin-dependent diabetes and depression. She noted that the serial endoscopic procedures, while intensive, were preferable to the systemic side effects of immunosuppression.

## Data Availability

The original contributions presented in the study are included in the article/supplementary material. Further inquiries can be directed to the corresponding author/s.
